# Multiomic analysis identifies a high-risk signature that predicts early clinical failure in DLBCL

**DOI:** 10.1038/s41408-024-01080-0

**Published:** 2024-06-20

**Authors:** Kerstin Wenzl, Matthew E. Stokes, Joseph P. Novak, Allison M. Bock, Sana Khan, Melissa A. Hopper, Jordan E. Krull, Abigail R. Dropik, Janek S. Walker, Vivekananda Sarangi, Raphael Mwangi, Maria Ortiz, Nicholas Stong, C. Chris Huang, Matthew J. Maurer, Lisa Rimsza, Brian K. Link, Susan L. Slager, Yan Asmann, Patrizia Mondello, Ryan Morin, Stephen M. Ansell, Thomas M. Habermann, Thomas E. Witzig, Andrew L. Feldman, Rebecca L. King, Grzegorz Nowakowski, James R. Cerhan, Anita K. Gandhi, Anne J. Novak

**Affiliations:** 1 Division of Hematology, Mayo Clinic, Rochester, MN USA; 2 Informatics and Predictive Sciences, , Bristol Myers Squibb, Summit, NJ USA; 3 Department of Quantitative Health Sciences Research, Mayo Clinic, Rochester, MN USA; 4 Informatics and Predictive Sciences, Celgene Institute for Translational Research Europe (CITRE), Seville, Spain; 5 Translational Medicine Hematology, Bristol Myers Squibb, Summit, NJ USA; 6 Division of Hematopathology, Mayo Clinic, Scottsdale, AZ USA; 7 Division of Hematology, University of Iowa, Iowa, USA; 8 Department of Quantitative Health Sciences Research, Mayo Clinic, Jacksonville, FL USA; 9 Genome Sciences Center, British Columbia Cancer Agency, Vancouver, BC Canada; 10 Department of Laboratory Medicine and Pathology, Mayo Clinic, Rochester, MN USA

**Keywords:** B-cell lymphoma, Translational research

## Abstract

Recent genetic and molecular classification of DLBCL has advanced our knowledge of disease biology, yet were not designed to predict early events and guide anticipatory selection of novel therapies. To address this unmet need, we used an integrative multiomic approach to identify a signature at diagnosis that will identify DLBCL at high risk of early clinical failure. Tumor biopsies from 444 newly diagnosed DLBCL were analyzed by WES and RNAseq. A combination of weighted gene correlation network analysis and differential gene expression analysis was used to identify a signature associated with high risk of early clinical failure independent of IPI and COO. Further analysis revealed the signature was associated with metabolic reprogramming and identified cases with a depleted immune microenvironment. Finally, WES data was integrated into the signature and we found that inclusion of *ARID1A* mutations resulted in identification of 45% of cases with an early clinical failure which was validated in external DLBCL cohorts. This novel and integrative approach is the first to identify a signature at diagnosis, in a real-world cohort of DLBCL, that identifies patients at high risk for early clinical failure and may have significant implications for design of therapeutic options.

## Introduction

While the majority of DLBCL patients are potentially cured after standard therapy, there remains a subset of patients who do not respond to front line treatment [1]. The approximate 70% of DLBCL patients treated with curative therapy in the frontline setting avoid retreatment, progression, relapse, or death within 24 months of diagnosis (termed event-free survival at 24 months or EFS24) have a good prognosis while the remaining 30% have a very poor outcome [2]. Using clinical factors we developed and validated the International Prognostic Index for EFS24 (IPI24) [3], which can be used at diagnosis for personalized risk prediction. Beyond clinical characteristics [4], molecular features associated with DLBCL prognosis include cell-of-origin (COO) [5, 6] and *MYC, BCL2*, or *BCL6* translocation status, or *MYC* “double hits (DH)” [7–9], with DH-DLBCL now considered as a distinct entity, High Grade B Cell Lymphoma (HGBCL) [10]. More recent molecular classification of DLBCL based on genomics, expression profiles, and tumor microenvironment has further refined our understanding of DLBCL heterogeneity and biologic underpinnings [11–18]. While these studies have advanced our understanding of DLBCL, none were designed to identify early failures (EFS24) after frontline standard of care therapy, which is of great interest for patient management and could provide biologic insight and identification of therapeutic targets. In addition, optimal utilization of novel treatment strategies such as chimeric-antigen receptor T cell (CAR T) or bispecific T-cell engager (BiTE) antibody therapy could benefit from identification of patients at high risk of early failure, as those therapies have not been shown to be related to currently defined molecular subtypes.

To identify a biologic signature of early clinical failure, we used next-generation sequencing (NGS) data generated on newly diagnosed (ndDLBCL) and relapsed/refractory DLBCL (rrDLBCL) tumors, combined with integrative computational approaches. Based on this signature, patients at diagnosis can be categorized into low, intermediate, or high-risk groups for early clinical failure (EFS24) and inferior overall event-free (EFS) and overall (OS) survival, independent of IPI, COO, and other known factors.

## Methods

### Study populations

The overall study design is shown in Fig. S1. We used clinical and NGS data from diagnostic tumors from 444 DLBCL patients from the University of Iowa and Mayo Clinic Lymphoma Specialized Program of Research Excellence (SPORE) Molecular Epidemiology Resource [19] (MER, *n* = 433) or from NCT00670358 (*n* = 11) [20], herein referred to as MER. All studies were performed in accordance with the Declaration of Helsinki. Patients provided written consent for use of clinical samples at study enrollment and this study was approved by the Mayo Clinic Institutional Review Board. Use of human Individual patient level data is shown in Table S1, all identifiers are coded, and full details are in Supplemental Methods. We also used NGS data from tumor samples at the time of relapse (rrDLBCL, any line of treatment and no overlap with ndDLBCL cohort), consented to the MER (*n* = 61), banked in the Mayo Lymphoma Biobank (waiver of consent) (*n* = 50), or consented to the CC-122-ST-001 clinical trial (*n* = 32, NCT01421524). NdDLBCL validation cohorts include those from the BCCA (EGAS00001002936), Duke (EGAD00001003600), and REMoDL-B (GSE117556) [18, 21, 22].

### DNA sequencing and analysis

For WES, we used paired tumor (FFPE) and germline (extracted from peripheral blood) DNA; sequencing was conducted at Expression Analysis, Inc (Durham, HC, USA), as described in Supplemental Methods. After quality control, WES data on 341 ndDLBCL were included. We also used existing WES data generated at Mayo on 19 additional ndDLBCL tumors, as well as previously analyzed WES data on Mayo DLBCL cases from Lohr et al (*n* = 16) and Hartert et al (*n* = 28) [23, 24]. The final analysis cohort included data from 404 ndDLBCL. Genes included for analysis are shown in Table S2. Mutation calls from the BCCA (*n* = 121) and Duke (*n* = 441) cohorts were provided by Dr. Morin, mutation calls from REMoDL-B (*n* = 400) were obtained from Sha et al. [22] Copy number analysis (CNA) was carried out using the Nexus Copy Number (Biodiscovery) software, detailed in Supplemental Methods [24–26]. Classification methods for LymphGen [13] and HMRN [14] and tools used for analysis are in Supplemental Methods.

### RNA sequencing and analysis

RNA was extracted from FFPE tissue sections and sequencing was performed at Expression Analysis, Inc as described in Supplemental Methods. Sample and RNA QC is shown in Fig. S2 for a final cohort of 321 ndDLBCL and 143 rrDLBCL cases with no overlapping cases. For validation, we used data from BCCA provided by Dr. Morin (*n* = 121); Duke, downloaded from EGA and processed in the Mayo Clinic Bioinformatics Core (*n* = 442); and REMoDL-B, downloaded and processed on the NCBI GEO website (*n* = 928) [18, 21, 22]. Data analyses, including the weighted gene correlation network analysis (WGCNA), Ecotyper [16] and LME [17] classification, COO, LymphProg [27], and differential gene expression analysis, are described in Supplemental Methods.

### Statistical analysis

EFS was defined as time from diagnosis to disease relapse/progression, retreatment, or death, and EFS24 was defined as EFS status at 24 months after diagnosis [2]. OS was defined as the time from diagnosis to death from any cause. EFS and OS were evaluated using Cox proportional hazards models and Kaplan Meier curves. EFS24 survival curves were truncated at 24 months. Differences in survival curves were evaluated using the log-rank test. For enrichment analysis of categorical variables, the Chi Square or Fisher’s exact test was used. Comparative analyses were carried out using either the Wilcoxon or Kruskal-Wallis test. A *P* < 0.05 was considered statistically significant unless otherwise stated. Multiple testing was performed as indicated in individual analysis. Analysis were performed using R and GraphPad Prism [28].

## Results

### Cohort description and performance of published molecular classifiers for early clinical failure

**Fig. 1:**
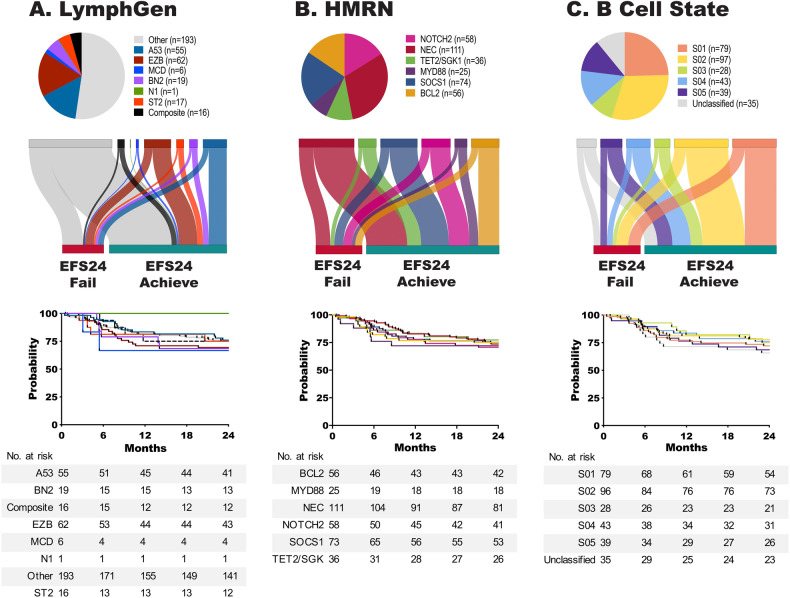
Current DLBCL classifiers do not discriminate early clinical failures. LymphGen (**A**), HMRN (**B**), and EcoTyper B Cell State (**C**) classification of ndDLBCL. Pie charts (upper panel) show distribution of cases for each classifier. Sankey plots (middle panel) show the distribution of EFS24 fail or achieve cases for each classifier. Kaplan−Meier analysis (lower panel) of EFS24 for each classifier, LymphGen *P* = 0.96, HMRN *P* = 0.98, and B Cell State *P* = 0.73.

A summary of available molecular and genetic features on the 444 ndDLBCL is shown in Fig. S3A. The median age at diagnosis was 64.5 years, 57% were male, and all were treated with immunochemotherapy; full clinical details are summarized in Fig. S3B. During a median follow-up time of 82.8 months (for living patients), 168 (37.8%) had an event and 112 (25.2%) failed to achieve EFS24. Those failing EFS24 had a median survival of only 18 months compared to 182 months for those who achieved EFS24. While some DLBCL molecular classifiers have been associated with prognosis, none were designed to discriminate early clinical failures. In our cohort, COO(5) (overall, *P* = 0.05, compared to GCB, ABC HR, 1.63 [95% CI 1.08–2.45] *P* = 0.019, and Unclassified HR, 1.50 [95% CI 0.84–2.67] *P* = 0.169), and DH FISH (DH FISH + HR, 1.93 [95% CI 0.96–3.86] *P* = 0.064), showed nominal associations with EFS24 in expected directions (Fig. S4A, B). The recently developed DLBCL molecular classifiers HMRN, LymphGen, and EcoTyper B Cell State showed expected distributions (Fig. 1A−C top panel), but were not associated with EFS24 (Fig. 1A−C, lower panel) and EFS24 failures spread across groups within each classifier as shown in Sankey plots (Fig. 1A−C, middle panel top). The genomic classification based on Chapuy et al was only available on 41 cases and therefore was not further analyzed [12].

### WGCNA analysis of DLBCL

**Fig. 2:**
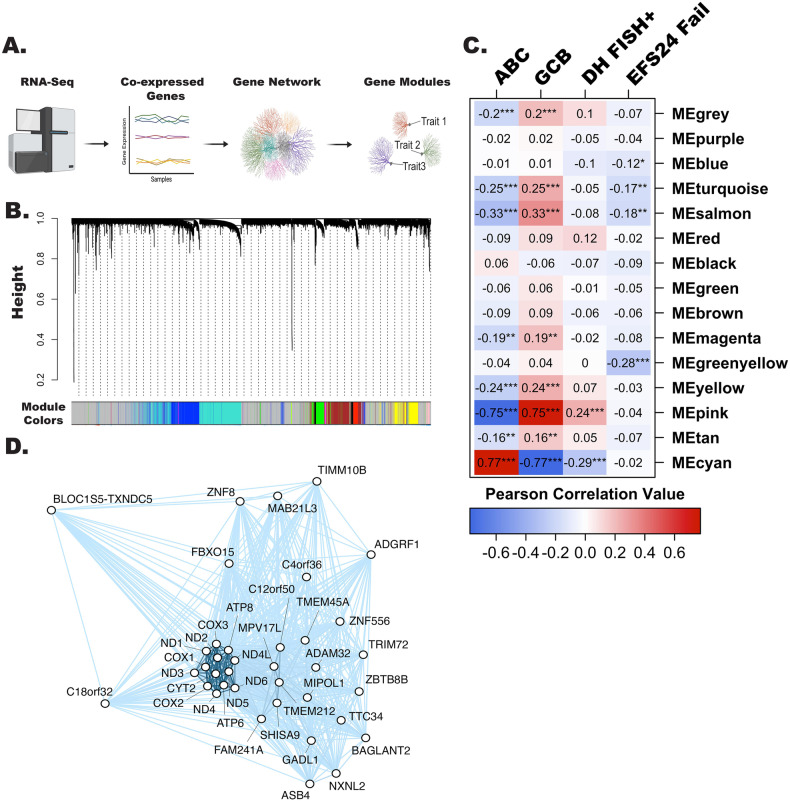
WGCNA analysis identifies biological modules associated with DLBCL clinical traits. **A** Schematic representation of WGCNA analysis workflow, created with BioRender.com. **B** Cluster dendrogram showing the 15 identified modules defined by color. The gray module consists of genes that could not be assigned to a co-expression module. **C** Correlation of individual WGCNA modules with selected traits (COO of ABC or GCB, *n* = 279, DH-FISH *n* = 252, and EFS24, *n* = 321) was performed using Pearson correlation, *P < 0.05, **P < 0.001 and ***P < 0.0001. **D** Correlation network representation of greenyellow module genes (*n* = 37) analyzed using the igraph R package.

Because the existing molecular classifiers were at best weak predictors of EFS24, we analyzed the RNA-seq data from our ndDLBCL cohort (*n* = 321) using WGCNA [29], a method for identifying biologic networks, or gene modules, by using pairwise correlations between variables (Fig. 2A). Unsupervised hierarchical clustering, followed by branch cutting, identified 15 modules with a range of 17 to 2685 genes (Fig. 2B). EFS24 as well as COO and DH characteristics were correlated with individual gene modules (Fig. 2C). The strongest positive correlations were observed for the cyan module (which included IRF4) with ABC and the pink module (which included BCL6) with GCB (Table S3). The pink module also showed strong correlation with DH FISH. Genes in the pink and cyan modules are in Table S3. As proof of principle for the utility of WGCNA, we calculated the eigengene gene score for each patient for the two COO-associated modules and found that the scores correlated well with their COO call (Fig. S5). The greenyellow module had the strongest correlation with EFS24 failure (*r* = −0.28), and was selected for further investigation. Genes (*n* = 37; Table S3) in this module had a negative correlation with EFS24 failure, suggesting that downregulation of their expression may be associated with early clinical failure (Fig. 2C). Figure 2D shows the tightly interconnected correlation network for the 37 genes in the greenyellow module.

### Generation of a high risk EFS24 failure gene signature

**Fig. 3:**
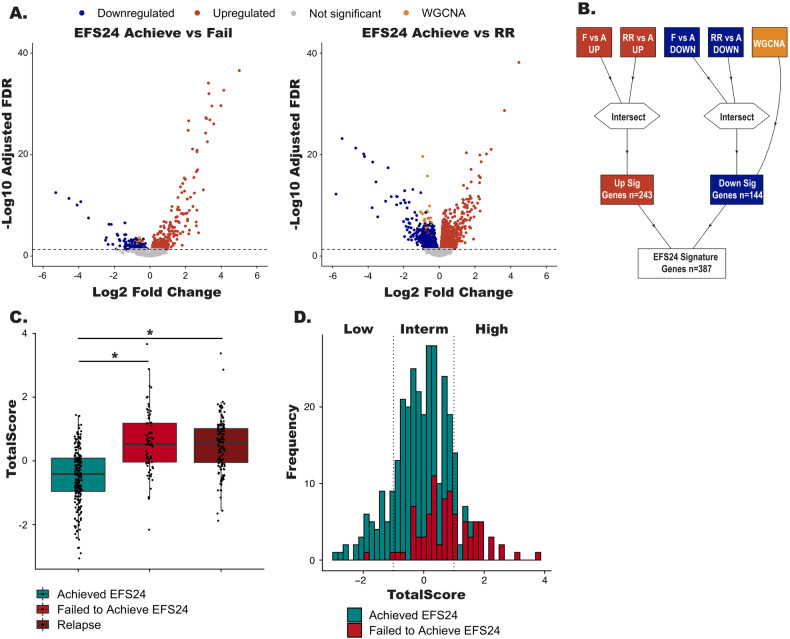
Generation of the RNA risk signatures. **A** Volcano plots showing differentially expressed genes (FDR < 0.05) between EFS24 achieve vs fail or EFS24 achieve vs rrDLBCL cases. Red dots represent upregulated genes, blue dots represent downregulated genes, gold dots represent genes identified in the WGCNA analysis, and gray dots are non-significant genes. **B** Schematic representation of how the 387 gene risk signature was generated. **C** Boxplots TotalScores for the risk signature foreach patient. *P < 0.05. **D** Distribution of the scaled Totalscores. Vertical lines represent +/- standard deviation which groups the scaled Totalscores samples into high, low and intermediated risk cases.

As the WGCNA analysis was unsupervised, we conducted complementary analyses that trained on the EFS24 endpoint using RNA-seq data from both nd- and rrDLBCL cases. We selected protein coding genes that were (1) differentially expressed (FDR < 0.05, *n* = 779 genes) between cases who failed (*n* = 84) and achieved (*n* = 237) EFS24; and (2) differentially expressed (FDR < 0.05, *n* = 3640 genes) between cases who achieved EFS24 (*n* = 235) and rrDLBCL cases (*n* = 143, Fig. 3A). Next, genes common to both analyses were intersected and added to the 37 genes from the WGCNA analysis, ultimately defining a 387 gene signature associated with EFS24 failures and relapsed disease (Fig. 3B and Table S4). To score each patient for the gene signature, the R tool singscore was used, which generates a totalscore for all 387 up- and downregulated genes (Fig. 3C) [30]. Patients who achieved EFS24 had a lower totalscore (median = 0.108), while patients who failed EFS24 (median = 0.165) or who had rrDLBCL (median = 0.168) had significantly higher totalscores. Next, we divided the cases into three groups based on the distribution of their totalscore by using a cut point of +/- one standard deviation (Fig. 3D), which shows that EFS24 failures increase with higher scores. A heatmap of the 387 gene signature, herein referred to as the risk signature, for the low, intermediate, and high risk groups is shown in Fig. S6.

### RNA risk signature is associated with prognosis

**Fig. 4:**
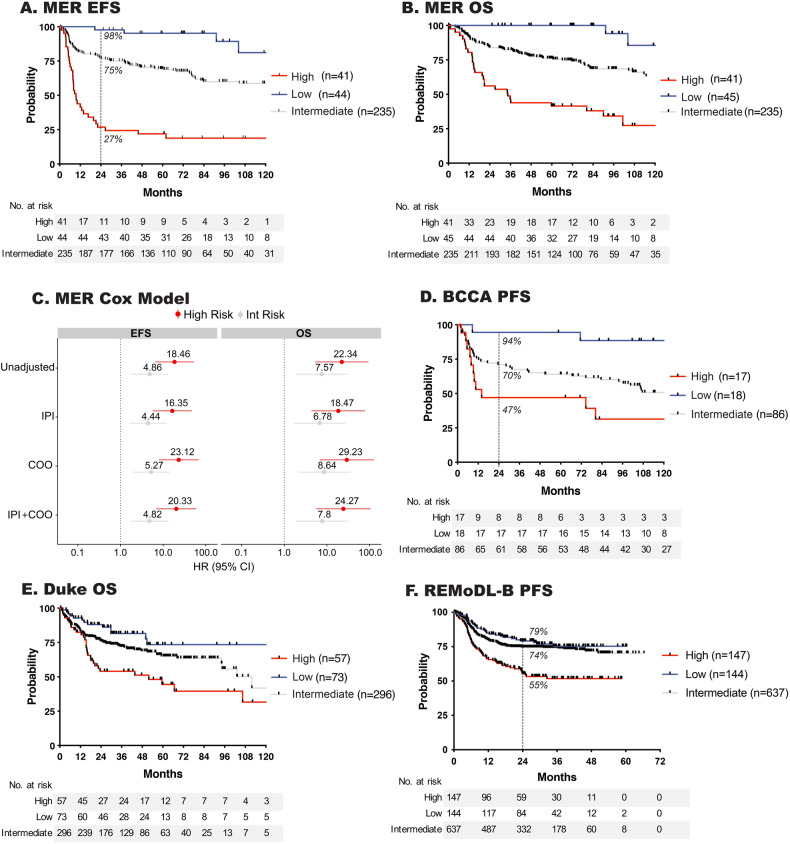
Outcome and clinical characteristics of risk signature groups. **A** Event free and (**B**) overall survival of MER ndDLBCL cases according to RNA risk signature classification. Cox model of high and intermediate risk signatures compared to low risk unadjusted or adjusted for IPI and/or COO, HR shown on image. **D**−**F** Validation of RNA risk signature association with outcome in the BCCA, Duke and REMoDL-B DLBCL cohorts. The hashed line and percentage shown on EFS curves indicate the percent of case achieving EFS24 in each risk group.

There was a strong association of the RNA signature score with EFS (Fig. 4A) and OS (Fig. 4B). Compared to low risk, patients with an intermediate or high risk signature had inferior EFS and OS, which did not attenuate after adjustment for COO and IPI (Fig. 4C). Furthermore, results held in analyses stratified on COO, IPI, after exclusion of HGBCL cases, and by treatment (Fig. S7A−G). The clinical features of cases in each risk group are shown in Table S5. Next, we attempted to validate our findings using gene expression data from cases with available outcome data from BCCA, Duke, and REMoDL-B (Fig. 4D−F). Compared to the low risk RNA signature group, patients with a high risk signature in BCCA (PFS, HR, 9.62 [95% CI 2.12–43.55] *P* = 0.003), Duke (OS, HR, 3.04 [95% CI 1.58–5.83] *P* = 0.001) and REMoDL-B (PFS, HR, 2.4 [95% CI 1.57–3.68] *P* < 0.001) had inferior outcomes.

### High risk cases are enriched with metabolic and TME signatures

**Fig. 5:**
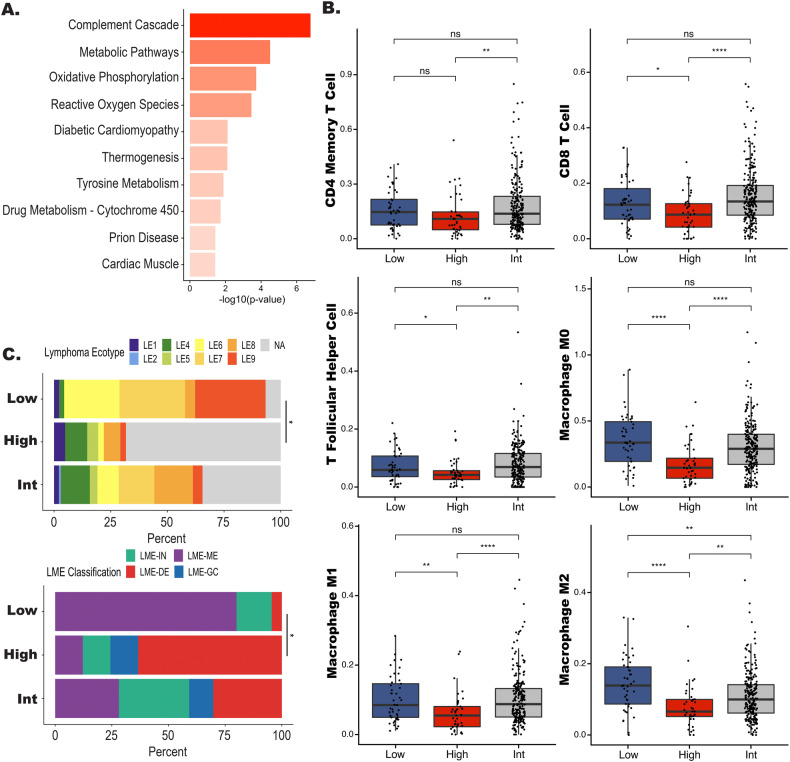
Pathway and TME characteristics of high risk signature DLBCL. **A** Bar plot displaying results from overrepresentation analysis for high risk cases. **B** Boxplots of individual cell populations identified by CIBERSORTX in the low, high, and intermediate risk groups. *P* values represent comparison between all three groups performed by a Kruskal–Wallis test and the line represents a *P < 0.05 between high and low risk groups performed by Wilcoxon test. **C** Bar plot showing the distribution of Lymphoma EcoType and LME classification in each risk group. The line represents a *P < 0.05 for the comparison of the number of NA or LME-Depleted between the high and low risk groups.

To better understand the biology underlying the RNA signature, we conducted additional in silico analyses. Using a 26 gene TME score (TME26) [31], we found a significant enrichment of TME negative cases in the high risk group (Table S5, *P* < 0.0001), suggesting the importance of overall cellular composition and TME biology in these tumors. To further explore which biological processes drive the high risk signature, we first performed pathway analysis. We first looked at all 3 RNA analyses individually, the WGCNA gene set, and the DEG analyses from Fig. 3A. As shown in Fig. S8A, oxidative phosphorylation and metabolic processes genes are represented in the greenyellow module. In the DEG analysis between EFS24 achieve vs EFS24 fail we again identified oxidative phosphorylation and metabolic processes (Fig. S8B). In the DEG analysis between EFS24 achieve and rrDLBCL, there were genes involved in mismatch repair, NF-κB, and inflammation (Fig. S8C). Next, we ran overrepresentation analysis on the 387 genes from our high risk signature and again identified genes that are involved in metabolic processes and oxidative phosphorylation (Fig. 5A). We then performed differential gene expression analysis between case categorized as high risk (*n* = 41) vs low risk (*n* = 45) and found that high risk cases were enriched for pathways related to oxidative phosphorylation, glycolysis, DNA replication, Myc targets, and BCR signaling. Supporting the TME26 finding, we also identified downregulation of immune related pathways (Fig. S8D).

To further expand on the TME findings and explore the immune composition of the high risk tumors we profiled the TME using computational prediction approaches to assess immune cell content. As shown in Fig. 5B using CibersortX, we found that there was a significant decrease in CD4 memory and CD8 T cells, T follicular helper (T_FH_), M0, M1, and M2 macrophages in the high risk group. These results were further supported when we examined the TME using the Lymphoma Ecotype or LME classifiers (Fig. S9), both of which identified a significant increase in LME depleted or unclassified TME Ecotype in the high cases vs low risk cases (Fig. 5C). Of note, EcoTyper and LME classification were not associated with EFS24 (Fig. S9).

### Integration of high risk signature with genetic features

**Fig. 6:**
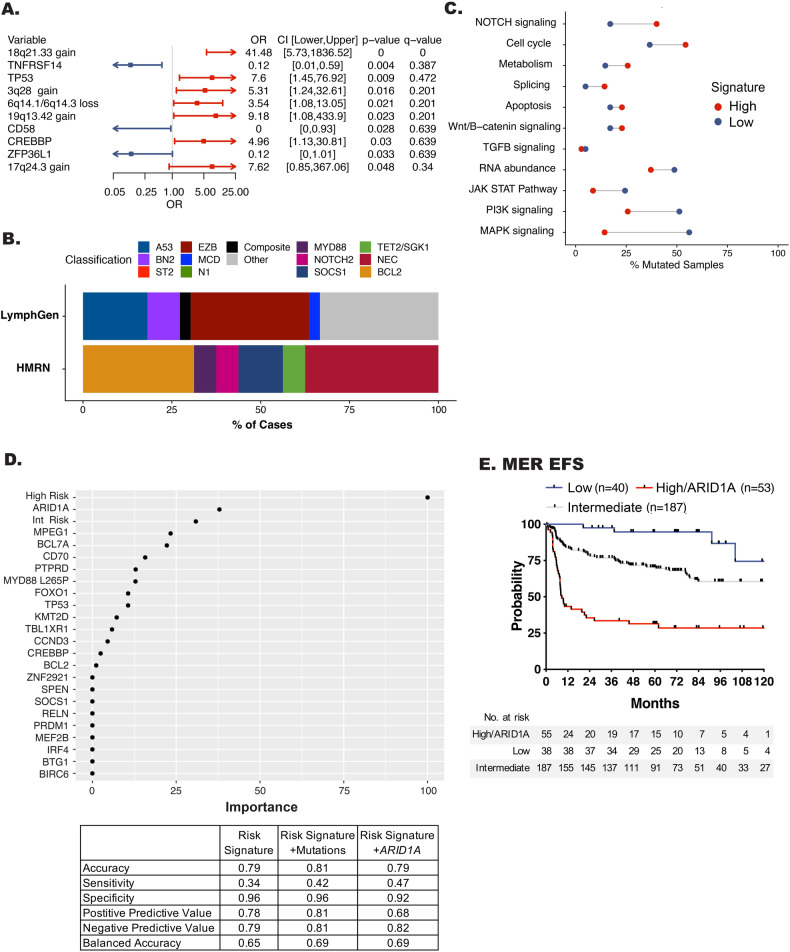
Genetic features of high risk DLBCL. **A** Forest plot showing enrichment of mutations and copy number events between the high and low risk groups. **B** Bar plot showing the distribution of LymphGen and HMRN classification in the high risk group. **C** Dot plot showing the percentage of samples which have mutations in the represented pathways. Red dots represent the percentage of samples in the high risk group while blue dots represent the percentage of cases in the low risk group. Shown pathways have at least a 1.3 fold increase or decrease between both groups. **D** Lasso regression model of the predictive value of the risk signatures alone (left panel) or with inclusion of mutations (right panel). Lasso metrics of the risk signature alone, with mutations, or with *ARID1A* are shown in the Table. **E** Kaplan−Meir curve showing event free survival of high risk DLBCL with the inclusion of *ARID1A* mutations.

To determine if our high risk signature was driven by unique genetic features, we used WES and OncoScan data to define their mutation and copy number landscape. Oncoplots for the mutation and copy number variants are shown in Fig. S10A, B. The high risk cases were significantly enriched for mutations in *TP53* and *CREBBP* as well as copy number alterations in 18q21.33 (*BCL2*), 3q28 (*BCL6*), 6q14 (*TMEM30A*), 19q13.42, and 17q24.3 when compared to low risk (Fig. 6A and Table S6). Enrichments by COO are shown in Fig. S10C. Classification of by LymphGen and HMRN (Fig. 6B and Fig. S10D) revealed that the high risk cases are spread across the molecular classifiers. While no significant enrichments were found in the high risk cases, we did see increased frequency of EZB, A53, and BN2, supporting the mutation and translocation enrichments for *BCL2*, *CREBBP*, *P53*, and *BCL6*. Because DLBCL is genetically heterogeneous and not dominated by single mutations, it is possible to miss the importance of less frequent variants that may play a role in aggressive biology. We therefore performed a pathway analysis using all genes previously reported to be mutated in lymphoma (*n* = 268) as well as the PanCancer gene list (*n* = 184) from maftools (Table S7) [11, 12, 18, 32, 33]. This analysis revealed an enrichment of mutations in genes related to Notch signaling, the cell cycle, splicing, and metabolism pathways, when compared to low risk (Fig. 6C). Conversely, there was enrichment of mutations in genes related PI3-kinase, Jak/Stat, and MAP-kinase in the low risk cases.

In our ndDLBCL cohort the high risk signature captured 36% of EFS24 failures. Therefore, we next wanted to determine if we could integrate genetic features with the risk signatures to further refine our ability to identify early failures (Fig. 6D). Using Lasso regression, we modeled the risk signatures alone or with the inclusion of mutations (frequency ≥5%, OR > 1, and *P* ≤ 0.15 in a Chi-square test for association with EFS24, *n* = 22, Table S2). As expected, the high risk signature alone strongly predicted EFS24 (Fig. 6D). Addition of mutations to the model found that *ARID1A* was associated with EFS24, and addition of *ARID1A* to the high risk signature, including patients with either an *ARID1A* mutation and/or the high risk signature, improved our model by increasing sensitivity and predicting both positive and negative outcomes more evenly (Fig. 6D). *ARID1A* mutations were also associated with EFS24 in the entire cohort (unadjusted, HR, 2.42 [95% CI 1.17–4.93] *P* = 0.014, adjusted for IPI, HR, 2.19 [95% CI 1.02–4.59] *P* = 0.04). Next, we categorized patients who had both RNA-seq and WES data as low, intermediate, or high risk and/or had an *ARID1A* mutation. Similar to prior analysis, compared to low risk the new integrated high risk signature was associated with EFS (HR, 13.53 [95% CI 4.84–37.84] *P* < 0.001) (Fig. 6E.) Overall, the integrated high risk signature captured 45% of EFS24 failure in the MER cohort. The association with poor outcome of integrated model including *ARID1A* was validated in independent data sets (Kaplan−Meier curves shown in Fig. S11A−C) from BCCA (PFS, HR 5.02, [95% CI 1.14–21.98] *P* = 0.03), and Duke (OS, HR 2.8, [95% CI 1.48–5.32] *P* = 0.001), and REMoDL-B (PFS, HR 2.65, [95% CI 0.85–3.19] *P* = 0.13).

**Fig. 7:**
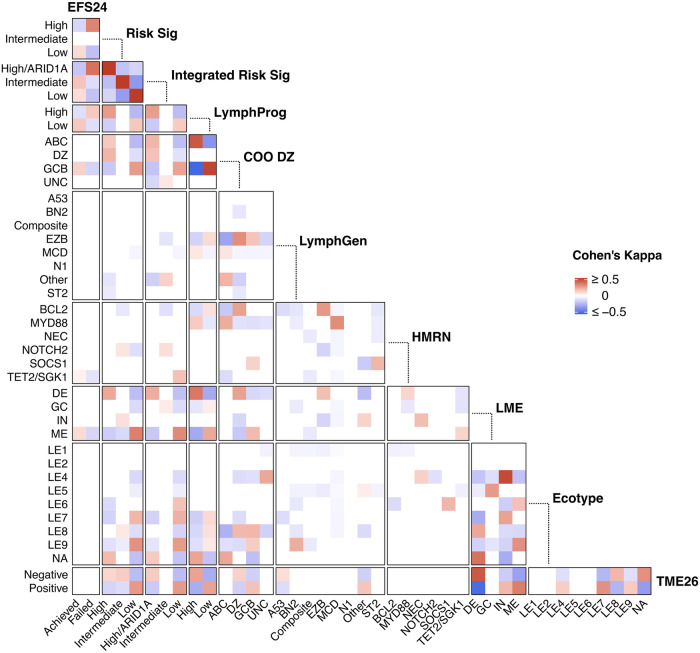
Agreement between DLBCL classifiers in MER DLBCL. To measure the agreement, or co-occurance, between individual classifiers (ie LympGen EZB with HMRN BCL2), Cohen’s kappa statistic was used. Agreements that had a 95% CI that did not span 0 in a positive (red) or negative (blue) direction are shown in the heatmap.

Recent advanced in the field have highlighted new classification models for DLBCL. Dark zone signature (DZsig), [34] which has recently been incorporated into COO classification and was previously referred to as double hit signature, identifies a subset of GCB and unclassified cases with poor outcomes. Additionally, Ren et all developed a gene expression score, LymphProg, that is a prognostic predictor for treatment outcomes [27]. We first looked at the gene sets for each classifier and found that our high risk signature shared 5 (1%) genes with DZ, and no genes with LymphProg, suggesting minimal overlap in signatures. To determine if our new high risk signature identified overlapping cases with either signature we first classified our samples as DZ or high/low for LymphProg. The co-occurrence between individual classifiers was quantified using Cohen’s kappa coefficient (κ), which tests for agreement between nominal variables, such as MCD with MYD88. As shown in Fig. 7, we measured agreement of our risk signatures with individual classifiers categories and compared the probabilities of each to identify overlapping samples. Positive agreements are shown in red and negative in blue. Cohen’s kappa values for all comparisons are shown in Table S8. This analysis found that cases captured by our integrated risk signature positively agreed with EFS24 failure (κ = 0.37) and LME DE (κ = 0.21), but also LymphProg (κ = 0.25), ABC (κ = 0.19), and DZ (κ = 0.16). This agreement was expected, as we found that our cases identified by the integrated risk signature were a mixture of ABC (56%), DZ (24%), GCB (18%), and unclassified (2%). The LymphProg high cases agreed most highly with ABC (κ = 0.43) and LME-DE (κ = 0.35), less so with EFS24 failure (κ = 0.14). This analysis also showed expected agreements across all comparisons including LME-IN and LE4 (κ = 0.47), both characterized by immune cell infiltration and inflammation, TME26 negative and LME-DE (κ = 0.43), both lacking immune cells, MCD with MYD88 (κ = 0.3), both of which harbor *MYD88* mutations, and EZB with BCL2 (κ = 0.27), both classified by mutations in *BCL2* and *EZH2*. Together, these data suggests that our new risk signature is unique and identifies high risk cases across existing DLBCL classifiers.

## Discussion

In recent years, several molecular and immune classification systems have been developed to subgroup DLBCL, which has advanced the understanding of biological pathways driving this disease. However, our results suggest that these known clusters fail to identify patients with an early clinical failure, limiting their use in clinical decision making at diagnosis. Using a multiomic approach on a highly annotated cohort of ndDLBCL, we describe a risk signature that captured patients at risk of early failure and poor overall outcomes, and further in silico analysis suggests the aggressive biology is defined by metabolic expression profiles and depleted tumor microenvironments. In a simultaneous analysis of these data, we performed an unsupervised analysis on transcriptomics features from ndDLBCL patients and identified 7 clusters, one called A7 (Aggressive lymphoma 7) with poor prognosis and defined by ABC COO, low TMA, and high myc expression [35]. Interestingly, our high risk gene expression signature shared overlap with only 17% of A7 cases, suggesting that unique aggressive features were detected by each approach.

Our findings that high risk DLBCL tumors were driven by a metabolic signature are supported by prior gene expression studies where Monti et al. [36] identified a subgroup of DLBCL, OxPHOS-DLBCL, defined by a dysregulation of genes belonging to the mitochondrial oxidative phosphorylation pathway (OxPhos) [36] with further analysis suggesting that those tumors develop an independent nutrition mechanism [37]. However, the association of OxPHOS-DLBCL with outcome has not been fully explored. The metabolic shift being detected by our high risk signature may be consistent with the “Warburg Effect” [38] a well know mechanism of cancer progression that is often associated with aggressive disease. As recently reviewed by De Martino et al. [39], metabolic alterations associated with malignant transformation and tumor progression can influence tumor-infiltrating immune cells, which may explain our findings that high risk tumors with metabolic signatures have a depleted TME.

There is a growing literature suggesting that a lack of TME involvement has a negative impact on outcome [16, 17], Kotlov et al., reported that the highest number of non-responders on standard chemotherapy where classified as TME depleted [17]. Our high risk signature is unique in that it simultaneously captures cases with both metabolic and TME dysregulation, allowing for early capture of aggressive DLBCL with potentially heterogenous biologic programs. Although, we recognize that the signature genes do not clearly represent a specific TME signature, rather, we would hypothesize that the metabolic signature detected by the genes occurs early in tumor development with the TME alterations arising as the tumor progresses.

Several studies have attempted to identify the prognostic value of single genetic alterations [40, 41], yet there is little consensus between studies. However, our data do align with previous findings on *TP53* alterations, which have been shown to be prognostic of inferior survival in DLBCL.[41–44] Mutations in*TP53* were found to be associated with EFS24 in the entire cohort (*n* = 404, unadjusted, HR, 1.8 [95% CI 1.02–3.13] *P* = 0.04, adjusted for IPI, HR, 1.93 [95% CI 1.07–3.45] *P* = 0.03), but did not increase the predicative ability of our signature, most likely because the high risk signature already identified *TP53* mutated cases. Because *TP53* mutations are well known to be associated with metabolic rewiring and chemoresistance, we hypothesize it may play an important role in driving the metabolic signature identified in our study [44–48]. Beyond *TP53*, our analysis highlights a role for *ARID1A*, a member of the SWI/SNF complex, in aggressive disease biology. *ARID1A* mutations have been implicated in both tumor suppression and tumor initiation in many malignancies, including DLBCL [49, 50]. In addition to being an important chromatin modifier, *ARID1A* is involved in double strand break repair, homologous recombination, and mismatch repair pathways [51–55]. *ARID1A* can also directly bind *TP53* to enhance its activity [56], thus loss of *ARID1A* may act like a tumor suppressor and have a negative prognostic impact even in the absence of *TP53* alterations. *ARID1A* truncating mutations are a defining feature of the LymphGen EZB genetic subtype, which is enriched for GCB DLBCL and characterized by epigenetic dysregulation [13]. EZB is also defined by mutations in *CREBBP* and alterations in *BCL2*, both of which are enriched in our high risk cases. The ability of *CREBBP* to modulate the TME through downregulation of MHC expression [57] combined with the potential for *ARID1A* to drive cell proliferation may be an important mechanism that drives high risk DLBCL. This is further supported by recent findings by Barisic et al. [58] suggesting that *ARID1A* mutations may be linked to accelerated transformation of follicular lymphoma to DLBCL.

Moving forward, our signature and proposed classification approach (Fig. S11E) may have important clinical implications. While not a primary focus of this manuscript, our risk classifier identified cases with a low risk of having an early event. This may be a subgroup of patients that will benefit from standard of care treatment with R-CHOP and may be spared from use of more toxic or expensive therapies. Identification of cases at diagnosis with our high risk signature could select patients appropriate for clinical trials evaluating frontline CAR-T or CD20 x CD3 bispecific antibodies + R-CHOP, both of which are currently enrolling. These therapies have demonstrated efficacy in patients resistant to chemotherapy in the relapsed setting, though biomarkers of response and resistance, including molecular alterations, remains limited for these newer therapies. Earlier identification of these patients at diagnosis could allow for sooner CAR-T manufacturing reducing the percentage of patients with progression prior to receiving CAR-T, a key barrier to this therapy. We also identified several important biological pathways that may be directly targetable. Pre-clinical studies have shown that cell lines with ARID1A mutations are sensitive to EZH2 inhibitors [59], such as tazemetostat, which are currently approved for the treatment of FL and are in clinical trial development for DLBCL. Lastly, a growing preclinical literature points to the possibility of targeting cancer cell metabolism to achieve immunostimulatory effects that could be maximized with combined immunotherapy.

In summary, we used integrative computational approaches to identify a multiomic signature of early clinical failure. Our signature captures important clinical and pathological characteristics, individual molecular alterations, and biological pathways in one signature for patient stratification at diagnosis which may be used inform clinical management.

### Supplementary information

Supplemental Material

Table S1

Table S2

Table S3

Table S4

Table S5

Table S6

Table S7

Table S8

## Data Availability

The RNA and DNA sequencing data generated in this study are publicly available in dbGAP, accession phs003634v1.

## References

[1] Coiffier B, Sarkozy C (2016). Diffuse large B-cell lymphoma: R-CHOP failure-what to do?. Hematol Am Soc Hematol Educ Program.

[2] Maurer MJ, Ghesquieres H, Jais JP, Witzig TE, Haioun C, Thompson CA (2014). Event-free survival at 24 months is a robust end point for disease-related outcome in diffuse large B-cell lymphoma treated with immunochemotherapy. J Clin Oncol.

[3] Maurer MJ, Jais JP, Ghesquieres H, Witzig TE, Hong F, Haioun C (2016). Personalized risk prediction for event-free survival at 24 months in patients with diffuse large B-cell lymphoma. Am J Hematol.

[4] Shipp MA, Harrington DP, Anderson JR, Armitage JO, Bonadonna G, Brittinger G (1993). A predictive model for aggressive non-Hodgkin’s lymphoma. The International Non-Hodgkin’s Lymphoma Prognostic Factors Project. N. Engl J Med.

[5] Alizadeh AA, Eisen MB, Davis RE, Ma C, Lossos IS, Rosenwald A (2000). Distinct types of diffuse large B-cell lymphoma identified by gene expression profiling. Nature.

[6] Wright G, Tan B, Rosenwald A, Hurt EH, Wiestner A, Staudt LM (2003). A gene expression-based method to diagnose clinically distinct subgroups of diffuse large B cell lymphoma. Proc Natl Acad Sci USA.

[7] Barrans S, Crouch S, Smith A, Turner K, Owen R, Patmore R (2010). Rearrangement of MYC is associated with poor prognosis in patients with diffuse large B-cell lymphoma treated in the era of rituximab. J Clin Oncol.

[8] Rimsza LM, Leblanc ML, Unger JM, Miller TP, Grogan TM, Persky DO (2008). Gene expression predicts overall survival in paraffin-embedded tissues of diffuse large B-cell lymphoma treated with R-CHOP. Blood.

[9] Savage KJ, Johnson NA, Ben-Neriah S, Connors JM, Sehn LH, Farinha P (2009). MYC gene rearrangements are associated with a poor prognosis in diffuse large B-cell lymphoma patients treated with R-CHOP chemotherapy. Blood.

[10] Jaffe ES, Barr PM, Smith SM (2017). Understanding the New WHO Classification of Lymphoid Malignancies: why it’s important and how it will affect practice. Am Soc Clin Oncol Educ Book.

[11] Schmitz R, Wright GW, Huang DW, Johnson CA, Phelan JD, Wang JQ (2018). Genetics and pathogenesis of diffuse large B-Cell Lymphoma. N. Engl J Med.

[12] Chapuy B, Stewart C, Dunford AJ, Kim J, Kamburov A, Redd RA (2018). Molecular subtypes of diffuse large B cell lymphoma are associated with distinct pathogenic mechanisms and outcomes. Nat Med.

[13] Wright GW, Huang DW, Phelan JD, Coulibaly ZA, Roulland S, Young RM (2020). A probabilistic classification tool for genetic subtypes of diffuse large B cell lymphoma with therapeutic implications. Cancer Cell.

[14] Lacy SE, Barrans SL, Beer PA, Painter D, Smith AG, Roman E (2020). Targeted sequencing in DLBCL, molecular subtypes, and outcomes: a haematological malignancy research network report. Blood.

[15] Dubois S, Tesson B, Mareschal S, Viailly PJ, Bohers E, Ruminy P (2019). Refining diffuse large B-cell lymphoma subgroups using integrated analysis of molecular profiles. EBioMedicine.

[16] Steen CB, Luca BA, Esfahani MS, Azizi A, Sworder BJ, Nabet BY (2021). The landscape of tumor cell states and ecosystems in diffuse large B cell lymphoma. Cancer Cell.

[17] Kotlov N, Bagaev A, Revuelta MV, Phillip JM, Cacciapuoti MT, Antysheva Z (2021). Clinical and biological subtypes of B-cell lymphoma revealed by microenvironmental signatures. Cancer Discov.

[18] Reddy A, Zhang J, Davis NS, Moffitt AB, Love CL, Waldrop A (2017). Genetic and functional drivers of diffuse large B cell lymphoma. Cell.

[19] Cerhan JR, Link BK, Habermann TM, Maurer MJ, Feldman AL, Syrbu SI (2017). Cohort profile: the lymphoma Specialized Program of Research Excellence (SPORE) Molecular Epidemiology Resource (MER) cohort study. Int J Epidemiol.

[20] Nowakowski GS, LaPlant B, Macon WR, Reeder CB, Foran JM, Nelson GD (2015). Lenalidomide combined with R-CHOP overcomes negative prognostic impact of non-germinal center B-cell phenotype in newly diagnosed diffuse large B-Cell lymphoma: a phase II study. J Clin Oncol.

[21] Arthur SE, Jiang A, Grande BM, Alcaide M, Cojocaru R, Rushton CK (2018). Genome-wide discovery of somatic regulatory variants in diffuse large B-cell lymphoma. Nat Commun.

[22] Sha C, Barrans S, Cucco F, Bentley MA, Care MA, Cummin T (2019). Molecular high-grade B-Cell Lymphoma: defining a poor-risk group that requires different approaches to therapy. J Clin Oncol: Off J Am Soc Clin Oncol.

[23] Lohr JG, Stojanov P, Lawrence MS, Auclair D, Chapuy B, Sougnez C (2012). Discovery and prioritization of somatic mutations in diffuse large B-cell lymphoma (DLBCL) by whole-exome sequencing. Proc Natl Acad Sci USA.

[24] Hartert KT, Wenzl K, Krull JE, Manske M, Sarangi V, Asmann Y (2021). Targeting of inflammatory pathways with R2CHOP in high-risk DLBCL. Leukemia.

[25] Krull JE, Wenzl K, Hartert KT, Manske MK, Sarangi V, Maurer MJ (2020). Somatic copy number gains in MYC, BCL2, and BCL6 identifies a subset of aggressive alternative-DH/TH DLBCL patients. Blood Cancer J.

[26] Wang Y, Wenzl K, Manske MK, Asmann YW, Sarangi V, Greipp PT (2019). Amplification of 9p24.1 in diffuse large B-cell lymphoma identifies a unique subset of cases that resemble primary mediastinal large B-cell lymphoma. Blood Cancer J.

[27] Ren W, Wan H, Own SA, Berglund M, Wang X, Yang M (2023). Genetic and transcriptomic analyses of diffuse large B-cell lymphoma patients with poor outcomes within two years of diagnosis. Leukemia.

[28] Team RC. (2022). R: A language and environment for statistical computing.

[29] Langfelder P, Horvath S (2008). WGCNA: an R package for weighted correlation network analysis. BMC Bioinforma.

[30] Foroutan M, Bhuva DD, Lyu R, Horan K, Cursons J, Davis MJ (2018). Single sample scoring of molecular phenotypes. BMC Bioinforma.

[31] Risueño A, Hagner PR, Towfic F, Fontanillo C, Djebbari A, Parker JS (2020). Leveraging gene expression subgroups to classify DLBCL patients and select for clinical benefit from a novel agent. Blood.

[32] Pillonel V, Juskevicius D, Ng CKY, Bodmer A, Zettl A, Jucker D (2018). High-throughput sequencing of nodal marginal zone lymphomas identifies recurrent BRAF mutations. Leukemia.

[33] Sanchez-Vega F, Mina M, Armenia J, Chatila WK, Luna A, La KC (2018). Oncogenic signaling pathways in the cancer genome atlas. Cell.

[34] Alduaij W, Collinge B, Ben-Neriah S, Jiang A, Hilton LK, Boyle M (2023). Molecular determinants of clinical outcomes in a real-world diffuse large B-cell lymphoma population. Blood.

[35] Stokes ME, Wenzl K, Huang CC, Ortiz Estevez M, Maurer MJ, Stong N (2022). Biological features of a high-risk transcriptional molecular subtype in diffuse large B-cell lymphoma. Blood.

[36] Monti S, Savage KJ, Kutok JL, Feuerhake F, Kurtin P, Mihm M (2005). Molecular profiling of diffuse large B-cell lymphoma identifies robust subtypes including one characterized by host inflammatory response. Blood.

[37] Caro P, Kishan AU, Norberg E, Stanley IA, Chapuy B, Ficarro SB (2012). Metabolic signatures uncover distinct targets in molecular subsets of diffuse large B cell lymphoma. Cancer Cell.

[38] Warburg O (1956). On the origin of cancer cells. Science.

[39] De Martino M, Rathmell JC, Galluzzi L, Vanpouille-Box C. Cancer cell metabolism and antitumour immunity. Nat Rev Immunol. 2024; 10.1038/s41577-024-01026-4. Epub ahead of print. 10.1038/s41577-024-01026-4 PMC11365797 38649722

[40] Bolen CR, Klanova M, Trneny M, Sehn LH, He J, Tong J (2020). Prognostic impact of somatic mutations in diffuse large B-cell lymphoma and relationship to cell-of-origin: data from the phase III GOYA study. Haematologica.

[41] Xu-Monette ZY, Wu L, Visco C, Tai YC, Tzankov A, Liu WM (2012). Mutational profile and prognostic significance of TP53 in diffuse large B-cell lymphoma patients treated with R-CHOP: report from an International DLBCL Rituximab-CHOP Consortium Program Study. Blood.

[42] Rushton CK, Arthur SE, Alcaide M, Cheung M, Jiang A, Coyle KM (2020). Genetic and evolutionary patterns of treatment resistance in relapsed B-cell lymphoma. Blood Adv.

[43] Zenz T, Kreuz M, Fuge M, Klapper W, Horn H, Staiger AM (2017). TP53 mutation and survival in aggressive B cell lymphoma. Int J Cancer.

[44] Aas T, Børresen AL, Geisler S, Smith-Sørensen B, Johnsen H, Varhaug JE (1996). Specific P53 mutations are associated with de novo resistance to doxorubicin in breast cancer patients. Nat Med.

[45] Rossi D, Cerri M, Deambrogi C, Sozzi E, Cresta S, Rasi S (2009). The prognostic value of TP53 mutations in chronic lymphocytic leukemia is independent of Del17p13: implications for overall survival and chemorefractoriness. Clin Cancer Res.

[46] Bergamaschi D, Gasco M, Hiller L, Sullivan A, Syed N, Trigiante G (2003). p53 polymorphism influences response in cancer chemotherapy via modulation of p73-dependent apoptosis. Cancer Cell.

[47] Tung MC, Lin PL, Wang YC, He TY, Lee MC, Yeh SD (2015). Mutant p53 confers chemoresistance in non-small cell lung cancer by upregulating Nrf2. Oncotarget.

[48] Dhayat SA, Mardin WA, Seggewiß J, Ströse AJ, Matuszcak C, Hummel R (2015). MicroRNA profiling implies new markers of gemcitabine chemoresistance in mutant p53 pancreatic ductal adenocarcinoma. PLoS One.

[49] Mullen J, Kato S, Sicklick JK, Kurzrock R (2021). Targeting ARID1A mutations in cancer. Cancer Treat Rev.

[50] Xu S, Tang C (2021). The role of ARID1A in tumors: tumor initiation or tumor suppression?. Front Oncol.

[51] Watanabe R, Ui A, Kanno S, Ogiwara H, Nagase T, Kohno T (2014). SWI/SNF factors required for cellular resistance to DNA damage include ARID1A and ARID1B and show interdependent protein stability. Cancer Res.

[52] Shen J, Ju Z, Zhao W, Wang L, Peng Y, Ge Z (2018). ARID1A deficiency promotes mutability and potentiates therapeutic antitumor immunity unleashed by immune checkpoint blockade. Nat Med.

[53] Samartzis EP, Noske A, Dedes KJ, Fink D, Imesch P (2013). ARID1A mutations and PI3K/AKT pathway alterations in endometriosis and endometriosis-associated ovarian carcinomas. Int J Mol Sci.

[54] Yamamoto S, Tsuda H, Takano M, Tamai S, Matsubara O (2012). Loss of ARID1A protein expression occurs as an early event in ovarian clear-cell carcinoma development and frequently coexists with PIK3CA mutations. Mod Pathol.

[55] Shen J, Peng Y, Wei L, Zhang W, Yang L, Lan L (2015). ARID1A deficiency impairs the DNA damage checkpoint and sensitizes cells to PARP inhibitors. Cancer Discov.

[56] Guan B, Gao M, Wu CH, Wang TL, Shih IeM (2012). Functional analysis of in-frame indel ARID1A mutations reveals new regulatory mechanisms of its tumor suppressor functions. Neoplasia.

[57] Green MR, Kihira S, Liu CL, Nair RV, Salari R, Gentles AJ (2015). Mutations in early follicular lymphoma progenitors are associated with suppressed antigen presentation. Proc Natl Acad Sci USA.

[58] Barisic D, Chin CR, Meydan C, Teater M, Tsialta I, Mlynarczyk C (2024). ARID1A orchestrates SWI/SNF-mediated sequential binding of transcription factors with ARID1A loss driving pre-memory B cell fate and lymphomagenesis. Cancer Cell.

[59] Bitler BG, Aird KM, Garipov A, Li H, Amatangelo M, Kossenkov AV (2015). Synthetic lethality by targeting EZH2 methyltransferase activity in ARID1A-mutated cancers. Nat Med.

